# *GALNT14*-rs9679162 Genotypes Predict Post-immunotherapy Side Effect and Survival in Patients with Hepatitis B Virus-related Hepatocellular Carcinoma

**DOI:** 10.7150/jca.133473

**Published:** 2026-06-10

**Authors:** Po-Ting Lin, Wei Teng, Wei-Ting Chen, Yu-De Chu, Chung-Wei Su, Yi-Chung Hsieh, Chen-Chun Lin, Yung-Chang Lin, Chun-Yen Lin, Chau-Ting Yeh, Shi-Ming Lin

**Affiliations:** 1 Department of Gastroenterology and Hepatology, Chang Gung Memorial Hospital, Linkou branch, No. 5, Fuxing St., Guishan Dist., Taoyuan City, 333, Taiwan.; 2 College of Medicine, Chang Gung University, No.259, Wenhua 1st Rd., Guishan Dist., Taoyuan City 333, Taiwan.; 3 Graduate Institute of Clinical Medical Sciences, College of Medicine, Chang Gung University, No.259, Wenhua 1st Rd., Guishan Dist., Taoyuan City 333, Taiwan.; 4 Liver Research Center, Linkou Chang Gung Memorial Hospital, No. 5, Fuxing St., Guishan Dist., Taoyuan City, 333, Taiwan.; 5 Department of Gastroenterology and Hepatology, New Taipei Municipal Tucheng Hospital, No.6, Sec.2, Jincheng Rd., Tucheng Dist., New Taipei City 236, Taiwan.; 6 Department of Hematology-Oncology, Chang Gung Memorial Hospital, Linkou branch, No. 5, Fuxing St., Guishan Dist., Taoyuan City, 333, Taiwan.; 7 Institute of Stem Cell and Translational Cancer Research, Linkou Chang Gung Memorial Hospital, No. 5, Fuxing St., Guishan Dist., Taoyuan City, 333, Taiwan.

**Keywords:** hepatocellular carcinoma (HCC), progression-free survival (PFS), rs6752303, side effects, single nucleotide polymorphism (SNP)

## Abstract

**Introduction:**

Genomic alterations have been reported to correlate with patients' response to immune checkpoint inhibitor (ICI) therapy in hepatocellular carcinoma (HCC). The present study aimed to examine whether single nucleotide polymorphism (SNP) is associated with ICI treatment-related side effects and progression-free survival (PFS) in HCC patients.

**Methods:**

This retrospective study included 96 patients with HBV-related HCC receiving ICI (atezolizumab plus bevazizumab) therapy between 2020 and 2023. Five SNPs derived from a previous genome-wide association study linking to chemotherapy responses in HCC patients were included. Their predictive values for PFS and side effects were examined.

**Results:**

After ICI therapy, patients with *GALNT14*-rs9679162 “GG” genotype (median PFS = 19.7 months, 95% confidence interval [CI]: 12.3-35.7; p = 0.032) had a longer PFS, whereas *GALNT14*-rs675230, *BMP7*-rs6025211, *WWOX*-rs13338697 or *WWOX*-rs13333314 were not associated with PFS (all p > 0.05). *GALNT14*-rs9679162 “GG” genotype (odds ratio [OR] = 0.298, 95% CI: 0.096-0.926; p = 0.036) was also associated with a decreased risk of post-treatment high-grade aspartate aminotransferase (AST) elevation. Multivariate analysis showed that *GALNT14*-rs9679162 “GG” genotype (hazard ratio [HR] = 0.433, 95% CI: 0.212-0.886; p = 0.022) and high initial albumin-bilirubin (ALBI) grade (HR = 2.053, 95% CI: 1.153-3.590; p = 0.014) were independently associated with PFS.

**Conclusions:**

Genetic variant of an SNP, *GALNT14*-rs9679162, predicts post-treatment PFS and side effect in HBV-related HCC patients receiving ICIs therapy.

## Introduction

Hepatocellular carcinoma (HCC), the third leading cause of cancer-related deaths in the world, has been referred to as a highly devastating malignancy, with a low 5-year survival rate of about 18% 1. Although the management of HCC has significantly changed over the last few years owing to improved patient stratification and introduction of novel therapies, it remains debatable regarding which treatment should be considered as 'standard therapy' for HCC cases 2.

The immune checkpoint inhibitors (ICIs), including atezolizumab and bevacizumab, are currently being used as the first-line therapy in patients with HCC 3. Fin et al. 4 have revealed that atezolizumab combined with bevacizumab results in better overall and progression-free survival (PFS) outcomes than sorafenib in unresectable HCC. However, despite the encouraging results, immunotherapy does not guarantee a clinical benefit in all patients with HCC, and more than two-thirds of advanced stage patients do not respond to immunotherapy 5. In addition, although the toxicity profile of the ICIs (anti-PD-1 agents) seems favorable with about 10% of patients experiencing grade 3-4 adverse events, the risk of immune-mediated toxicities warrants strict vigilance concerning symptoms of colitis, pneumonitis, and especially hepatitis in patients with HCC 6.

A previous genome-wide association study attempting to identified SNPs linking to chemotherapeutic responses in advanced HCC has resulted in discovery of a few predictive markers. Of the two single nucleotide polymorphisms (SNPs) rs9679162 and rs6752303 flanking *GALNT14*, the TT genotype of *GALNT14*-rs9679162 was found to be a reliable marker for therapeutic outcome in advanced HCC patients treated with chemotherapy 7 or sorafenib 8. *WWOX* SNP (rs13338697) predicted the therapeutic efficacy of ADI-PEG 20 in patients with advanced HCC 9. A previous study reports that patients with HCC carrying the *GALNT14*-rs9679162 TT genotype have lower GALNT14 expression and favorable prognosis 10. They suggest that overexpression of GALNT14 may enhance hepatocarcinogenesis/progression and resistance to anticancer drugs via O-glycosylation at Ser161 of prohibitin-2 (PHB2) in the endoplasmic reticulum and/or cell membrane. Furthermore, GALNT14 genetic variants are correlated to HCC postoperative prognosis, and GALNT14 expression is associated with abundance of M2-macrophages in tumor microenvironment 11. A recent study indicates that patients with HBV-related HCC show better responses to immunotherapy 12. However, no study has currently investigated whether these SNPs are associated with ICI-related responses in HBV-positive advanced HCC patients. Further understanding of the genomic background of HCC is essential to overcome therapeutic challenges of ICIs so that clinicians can carefully select favorable patients to receive ICI therapy and, to advise unfavorable patients to choose other novel effective treatments. Therefore, the present study aimed to further determine the associations between these SNPs and ICI treatment-related side effects and survival in patients with HBV-related HCC.

## Materials and Methods

### Study design and patient selection

This retrospective study reviewed patients with HCC receiving ICIs (atezolizumab plus bevazizumab) therapy between 2020 and 2023. Exclusion criteria were: 1) Patients without HBV; 2) ICI treatment < 3 cycles; 3) No post-treatment image; 4) Curative HCC; and 5) Double cancers. After exclusion, 96 patients were included in the analysis (**Figure S1**).

Baseline demographic and clinical data collected from the medical records were age, sex, BCLC classification, microvascular invasion (MVI), portal vein invasion (VP) stage, up to 7 criteria 13, or up to 11 criteria 14, extra-hepatic metastasis (EHM), treatment with transarterial chemoembolization (TACE) and/or radiofrequency ablation (RFA), history of hepatitis B virus (HBV), neutrophil-to-lymphocyte ratio (NLR), platelets, albumin, alpha fetoprotein (AFP), γ-GT, alanine transaminase (ALT), albumin-bilirubin (ALBI) grade, and previous gastrointestinal drug. Patients were followed with hepatic ultrasonography and computed tomography (CT) every 3-6 months, and serum AFP and serum protein levels were assessed every month; these data were also extracted from the medical records.

### Main outcome measures assessment

Primary outcomes included overall survival (OS), PFS, treatment response, objective response rate (ORR), disease control rate (DCR) and a decrease of serum AFP of 20% from baseline in patients with ICI. The secondary outcomes were to determine the factors associated with post-treatment OS or post-treatment PFS. Treatment response evaluation was based on the modified Response Evaluation Criteria in Solid Tumors (mRECIST) 15. ORR was defined as the percentage of patients who achieve a response, which was either be complete response (CR; complete disappearance of lesions) or partial response (PR; reduction in the sum of maximal tumor diameters by at least 30%) 15. The DCR was defined as the percentage of patients who achieved a CR, PR, and stable disease, as reported previously 16. The OS rate was defined as the interval between ICI treatment and the date of death caused by HCC or the date of the last follow-up. PFS was defined as the interval between ICI therapy and the date of diagnosis of the first recurrence or the date of the last follow-up.

### Ethical considerations

The study protocol was approved by the Institutional Review Board (IRB) of our hospital (Approval number: 20240041B0) and was conducted in accordance with both the Declarations of Helsinki and Istanbul. In addition, the need for patients' informed consent was waived by the IRB because the study was as retrospective cross-sectional, observational study with expedited review. All medical records of patients diagnosed with HCC treated with the ICIs between 2020 and 2023 were reviewed (**Figure S1**).

### SNP genotyping

The genotyping of the SNPs was conducted following previously established procedures 10. In brief, genomic DNA isolated from blood samples was extracted using the QIAamp DNA mini kit (QIAGEN, Hilton, Germany), adhering to the manufacturer's provided instructions. Semi-nested polymerase chain reaction (PCR) was performed with the following primers: *GALNT14*-F1: 5'-TCACGAGGCCAACATTCTAG-3'; *GALNT14*-R1: 5'-TTAGA-TTCTGCATGGCTCAC-3'; *GALNT14*-R2: 5'-TCCCTCCTACTGAACCTCTCC-3'; *WWOX*-F1: 5'-ACTTCTGACAGCCATCCAGA-3'; *WWOX*-F2: 5'-ATCCTGCTAGCATGTTGACT-3'; *WWOX*-R2: 5'-ACTGTAGATGCCTTCCATCT-3'; Rs6025211-F1: 5'-ACATTCACAGAGAACTTGGC-3'; Rs6025211-R1: 5'-CAAGCAGTCCTTCCACCTTG-3'; Rs6025211-R2: 5'-AAAGTGCTGGGATTACAGG-T-3'. The amplified PCR products underwent gel purification using the EasyPrep Gel & PCR Extraction Kit (BIOTOOLS, New Taipei City, Taiwan).

### Side effects evaluation

Side effects were evaluated according to the National Cancer Institute Common Terminology Criteria for Adverse Events (CTCAE) version 5.0.

### Statistical analysis

Continuous variables were expressed as medians with corresponding range or 95% confidence interval (CI), and compared using Mann-Whitney U test. Categorical variables were expressed as number and percentage, and are compared using Fisher's exact test. The Kaplan-Meier method was used to compare survival times between the two genotypes, and survival curves were compared using the log-rank test. Cox regression analysis was performed to determine associations between demographic and clinical variables and PFS of patients with each genotype. Time-to-event was defined as “the follow-up period of event occurrence”. Logistic regression analysis was performed to determine associations between patients' demographic and clinical variables and post-treatment aspartate aminotransferase (AST) grade. A 2-sided value of p < 0.05 was established as statistical significance. Statistical analyses were performed using SPSS version 25.0 software (SPSS Inc., Chicago, IL, USA).

## Results

### Patient characteristics

The demographic and clinical characteristics of 96 patients with HCC are shown in **Table 1**, including 81 (84,4%) males and 15 (15.6%) females, with median age 60.7 (33.3-83.1) years. Median baseline scores for NLR, platelets, albumin, AFP, γ-GT and ALP were 3.9 (1.4-51.4), 176.0 (39.0-750.0) 103/μL, 3.8 (2.5-4.7) g/dL, 371.0 (2.0-211754.9) ng/mL, 127.0 (19.0-768.0) U/L and 119.0 (44.0-1283.0) U/L, respectively. The relative majority were BCLC stage C (74.0%), no MVI (51.0%), up-to-7 criteria (78.1%), up-to-11 criteria (57.3%), no EHM (53.1%), previous TACE (53.1%), no previous RFA (71.9%), history of HBV (100%), ALBI II or III (63.5%), and no previous GI drugs (83.3%). In addition, 23 (24%) patients were rs6752303 with TT genotype, 21 (21.9%) patients were *GALNT14*-rs9679162 with GG genotype, 14 (14.6%) patients were rs6025211 with TT genotype, 9 (9.4%) patients were rs13338697 with GG genotype, and 9 (9.4%) patients were rs13333314 with AA genotype.

### Outcomes between the two genotypes

**Figure 1:**
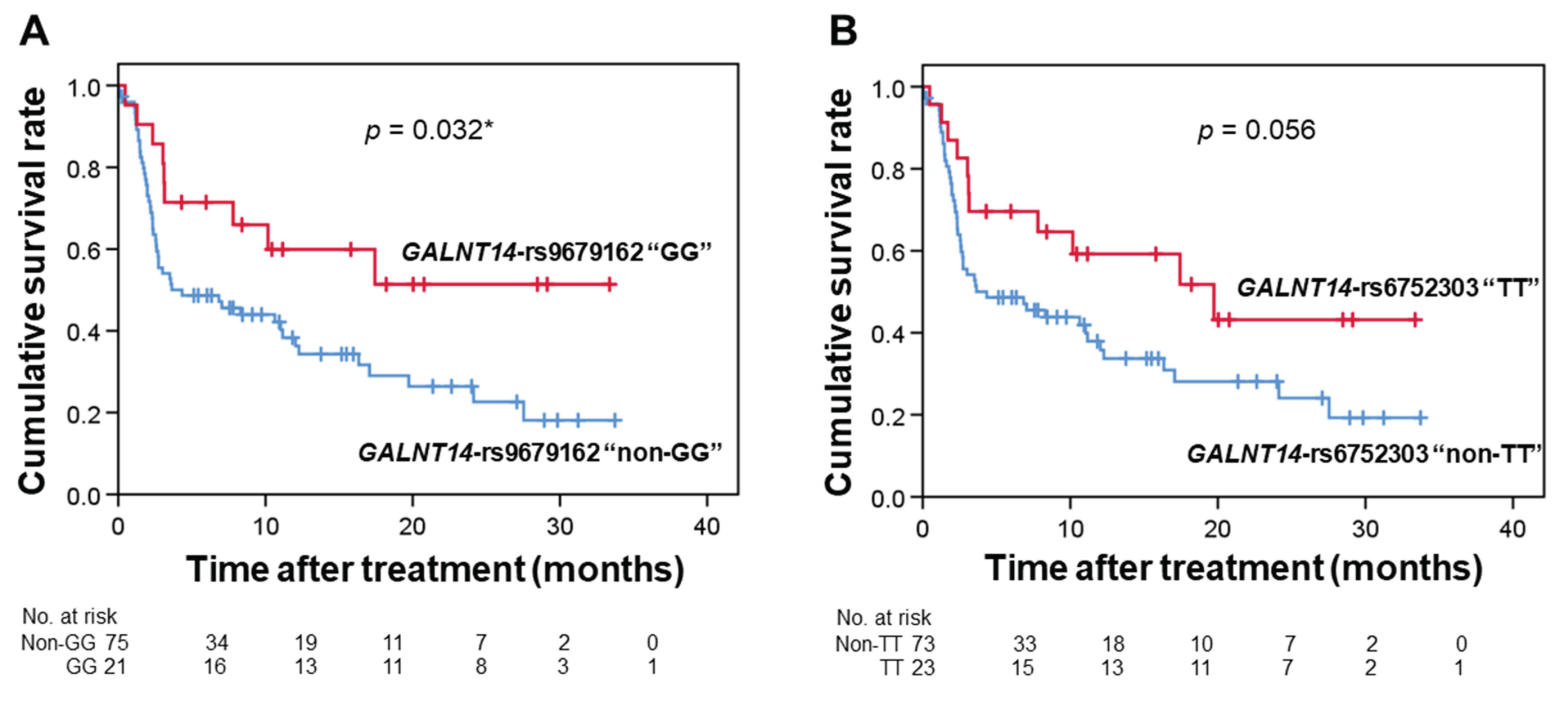
** Kaplan-Meier survival curves for HCC patients.** Kaplan-Meier survival curves for 96 HCC patients stratified by *GALNT14*-rs9679162 (A) and *GALNT14*-rs6752303 (B). *p<0.05.

After treatment, the median follow-up period in *GALNT14*-rs9679162 with GG and non-GG was 10.4 (1.4 ± 33.4) months and 7.8 (0.1-33.8) months, respectively (p > 0.05, **Table 2**). Patient with GG genotype had a higher median PFS than patients with non-GG genotype (GG vs non-GG genotypes: 19.7 [12.3-35.7] vs 4.3 [0-9.9] months; p = 0.032, **Table 2** and **Figure 1A**). Moreover, disease control rate slightly increased in patients with GG genotype (71.4%) when compared with those with non-GG genotype (50.7%) (p = 0.136, **Table 2**). During a similar median follow-up period, *GALNT14*-rs6752303 with TT also had a higher PFS than *GALNT14*-rs9679162 with non-TT, although it was not statistically different (TT vs. non-TT genotypes: 19.7 [5.2-34.3] vs 4.3 [0-9.8] months, p=0.056, **Table 2** and **Figure 1B**). Compared to patients with non-TT genotype (50.7%), disease control rate also slightly increased in patients GG genotype (69.6%) (p = 0.150, **Table 2**). In contrast, PFS and disease control rate were not significantly different between the genotypes of *BMP7*-rs6025211, *WWOX*-rs13338697 or *WWOX*-rs13333314 (all p > 0.05, **Table S1**).

### Associations between genotype and post-treatment side effects

Compared to *GALNT14*-rs9679162 with the non-GG genotype, *GALNT14*-rs9679162 with GG genotype had a higher proportion of post-treatment AST with a low grade (p = 0.034, **Table S2**). However, the distribution of other post-treatment side effects, including ALT, hypertension, proteinuria, hyperthyroidism, hypothyroidism, rash, fatigue, dizziness, myalgia, pruritus, diarrhea, HFSR, edema, fever, pneumonitis, colitis, abdominal pain, vomiting or nausea, GI bleeding, and GI bleeding prevention, was not significantly different between the two genotype groups (all p > 0.05, **Table S2**). Similarly, GALNT14 rs6752303 with TT genotype had a higher proportion of post-treatment AST with a low grade (p = 0.010, **Table S2**).

Subsequent multivariate logistic regression analysis showed that *GALNT14*-rs9679162 with GG (odds ratio [OR] = 0.298, 95% CI: 0.096-0.926; p = 0.036) was associated with a decreased risk of post-treatment AST high grade elevation (**Table 3**). However, age, sex, BCLC stage, MVI, out-to-7 criteria, EHM, and ALBI grade were not significantly associated with post-treatment AST grade (all p > 0.05, **Table 3**). Similarly, GALNT14 rs6752303 with TT (OR = 0.236, 95% CI: 0.077-0.721; p = 0.011) was associated with a decreased risk of post-treatment AST high grade elevation (**Table S3**).

### Factors associated with post-treatment PFS

In the multivariate Cox regression analysis, *GALNT14*-rs9679162 with GG (hazard ratio [HR] = 0.433, 95% confidence interval [CI]: 0.212-0.886; p = 0.022) was associated with a decreased risk of post-treatment disease progression, while ALBI grade II/III (HR = 2.053, 95% CI: 1.153-3.590; p = 0.014) was significantly associated with an increased risk of post-treatment disease progression (**Table 4**). However, sex, BCLC stage, MVI, out-to-7 criteria, out-to-11 criteria, EHM, 20% improvement of AFP, line of ICI treatment, post-treatment AST grade, and post-treatment HFSR grade were not significantly associated with post-treatment disease progression (all p > 0.05, **Table 4**).

### Side effects are associated with SNP *GALNT14*-rs9679162-affected post-treatment PFS

To determine whether *GALNT14*-rs9679162-related post-treatment AST involves in *GALNT14*-rs9679162-afftected post-treatment disease progression, we further evaluated the association between *GALNT14*-rs9679162 genotypes and post-treatment disease progression in different AST subgroups (grade 0 and grade ≥ 1) (**Table 5**). In low AST grade (grade 0) subgroup, multivariate Cox analysis showed that *GALNT14*-rs9679162 with GG genotype (HR = 0.350, 95% CI: 0.132-0.929; p = 0.035) was associated with a decreased risk of post-treatment disease progression; however, this association was not observed in high AST grade (grade ≥ 1) subgroup (p > 0.05). This indicated that post-treatment AST grade may be associated with *GALNT14*-rs9679162-related post-treatment disease progression.

In high AST grade subgroup, multivariate Cox analysis showed that high ALBI grade (grade II and III) (HR = 2.523, 95% CI: 1.122-5.672; p = 0.025) was associated with an increased risk of post-treatment disease progression; however, this association was not observed in low AST grade subgroup (p > 0.05). This indicated that post-treatment AST grade may be also associated with ALBI grade-related post-treatment disease progression.

## Discussion

The new key findings of the study included: 1) The *GALNT14*-rs9679162 “GG” genotype had a longer post-treatment PFS than did *GALNT14*-rs9679162 “non-GG” genotype in HBV-related HCC patients with ICIs; 2) The *GALNT14*-rs9679162 “GG” genotype was associated with a decreased risk of post-treatment AST high grade elevation; and 3) The severity of post-treatment AST grade is associated with *GALNT14*-rs9679162 SNP-affected post-treatment disease progression.

Despite the limited information on the GALNT14-rs9679162 genotype, existing data suggests that this SNP is located within the intron of the GALNT14 gene 17. SNP *GALNT14*-rs9679162 has been identified as a reliable indicator for a positive response to chemotherapy 7 or sorafenib 8 treatment in advanced HCC patients. According to the SNP findings in the present study, the frequency of the *GALNT14*-rs9679162 “GG” genotype in our population with HCC treated with ICIs (n = 96) was approximately 22.9%. In the present study, the *GALNT14*-rs9679162 genotype could predict a longer PFS following treatment with ICIs in HCC patients. Specifically, individuals with the *GALNT14*-rs967962 “GG” genotype exhibited a decreased risk of disease progression post-treatment with ICIs compared to those with the “non-GG” genotype. It has been shown that SNPs could modulate several cellular functions through alteration of mRNA secondary structure, modulation of RNA alternative splicing, and regulation of target-gene-translation efficiency 18. The GALNT14 expression is significantly associated with the adverse survival of patients with HCC 19. Taken together, it could be speculated that cancer cells in HCC patients with *GALNT14*-rs9679162 “GG” genotype are more sensitive to ICIs therapy. The expression of GALNT14 may be influenced by SNP *GALNT14*-rs9679162 via the potential underlying mechanisms mentioned above, resulting in increased sensitivity to ICIs therapy in HCC patients. The functional roles of the SNP *GALNT14*-rs9679162/GALNT14 expression/ICIs response axis in HCC need to be determined in the future.

The adverse effects of ICIs are generally mild, with approximately 10% of patients experiencing severe grade 3-4 events 6. Nevertheless, these inhibitors, such as CTLA-4 and PD-(L) 1 inhibitors, typically result in an increase in side effects, including elevated levels of AST 18, 20. Histologically, ICIs can lead to conditions like granulomatous hepatitis with fibrin deposition/central vein endotheliitis 20 or lobular non-granulomatous hepatitis 18. Notably, post-treatment AST-related liver dysfunction has been linked to poorer PFS in patients with advanced cancer following ICI (nivolumab or pembrolizumab) monotherapy 21. In our HBV-infected HCC patients receiving ICI (atezolizumab plus bevazizumab) therapy, it showed that post-treatment AST with a high grade was associated with a shorter PFS. More importantly, our data further demonstrated that the *GALNT14*-rs9679162 “GG” genotype was associated with a decreased risk of post-treatment AST with a high grade when compared with *GALNT14*-rs9679162 “non-GG” genotype. After stratifying by post-treatment AST grade, *GALNT*14-rs9679162 “GG” genotype associated with a decreased risk of post-treatment disease progression was only observed in subgroups with low post-treatment AST grade but not in subgroups with high post-treatment AST grade. According to these findings, it implicates that patient with *GALNT14*-rs9679162 “GG” genotype is more susceptible to ICI therapy, which might also reduce the occurrence of these side effects to further influence the post-treatment disease progression. For this aspect, we thus recommended that ICI therapy may be a suitable option for treating HCC patients with *GALNT14*-rs9679162 “GG” genotype. Moreover, post-treatment monitoring for AST-related side effect in HCC patients treated with ICI is necessary in the following follow-up.

The gene *GALNT14*, situated on chromosome 2, is a member of the polypeptide N-acetylgalactosaminyltransferase family. This family is involved in catalyzing protein O-glycosylation, and dysregulated GALNT expression has been linked to altered O-glycosylation patterns in various cancers 22, 23. Even though not much is known about how GALNT14 works in HCC, studies have shown that it helps cells divide and move around, and that blocking it makes cells more sensitive to anticancer drugs 10. Recently, Li et al. 9 have proposed that the downregulation of GALNT14 may induce ferroptosis by inhibiting the mTOR/EGFR pathway, subsequently reducing the protein levels of SLC7A11 and GPX4. Inhibition of ferroptosis has been associated with decreased AST levels and improved survival in drug-induced liver injuries 24. Teraoka 25 suggests that the unidentified immune activity in the tumor may enhanced the effect of ICI in the early phase, which results in liver injury due to T cell recognition and activity against antigens in healthy tissues. Based on the results of these findings and the present study, we speculated that SNP *GALNT14*-rs9679162 may influence the post-ICI PFS in HCC patients via GALNT14 expression-regulated ferroptosis/AST/liver injury axis. To provide improving treatment with ICIs and important clues on the mechanism of ICI-mediated toxicity and antitumor efficacy, further investigation is required to elucidate the mechanisms driving these associations.

Currently, age has not been utilized as a stratification variable that could influence safety and complications in the large-scale clinical trials of ICIs for HCC 26. However, adverse events (AE) and efficacy of immunotherapy in the older population may differ from those in younger patients 26. Compared to these findings, we noted that older age was associated with a decreased risk of post-treatment disease progression in HCC patients following ICI. Furthermore, this association was only observed in subgroups with high post-treatment AST grade but not in subgroups with low post-treatment AST grade, indicating that older age and severe post-treatment AST-related side effect could have a synergistic effect to reduce the risk of post-treatment disease progression. Mechanistically, a possible explanation is that older patients have a higher incidence of all-grade AEs after ICI therapy 27. Patients who experience a higher grade AE should have a higher T-cell activity and, hence, experience better antitumor outcomes than patients who experience a lower grade AE 28.

HCC patients with lower ALBI grades had better response to immunotherapy and prognosis 29. Similarly, the results of the present study also exhibited that an elevated ALBI grade (III + IV) was associated with an increased risk of post-treatment disease progression in HCC patients with ICI. Of note, we further demonstrated that this association was only observed in subgroups with high post-treatment AST grade but not in subgroups with low post-treatment AST grade, indicating that elevated ALBI grade and severe post-treatment AST-related side effect (liver dysfunction-related side effect) could have a synergistic effect to enhance the risk of post-treatment disease progression. For this finding, it may be because cirrhosis is the common concomitant liver disease in HCC patients, and an elevated ALBI grade is indicative of deteriorating liver function and more advanced cirrhosis 30. The presence of cirrhosis can facilitate tumor immune evasion and foster an immunosuppressive microenvironment. For instance, cirrhotic livers exhibit increased levels of extracellular matrix components, which can further inhibit anti-tumor immunity through the activation of transforming growth factor (TGF)-β 31. Moreover, liver fibrogenesis is stimulated by hepatic stellate cells, which may reduce lymphocyte infiltration and promote the proliferation of immunosuppressive cells 32.

The present retrospective study has several limitations. First, all patients were recruited from the same hospital and data were retrospective, which together may result in selection bias. Also, cross-sectional, observational study does not allow inferences of causality. Secondly, the sample size of the present study was relatively small, and further studies with a larger population are needed to verify the present findings. Lastly, the molecular mechanisms underlying the survival-associated SNP rs6752303/AST grade axis and the observed association call for direct biological experiments and future prospective study for functional validation.

## Conclusion

The present study identified a significant association between SNP *GALNT14*-rs9679162 and survival of HBV-related HCC patients received ICI therapy. The protective genotypes of this SNP may contribute to a better post-treatment PFS in an AST-mediated manner. These results may provide clinicians with some valuable information for risk stratification and treatment strategy development in the care of patients with HBV-related HCC undergoing ICIs therapy.

## Supplementary Material

Supplementary tables.

## Figures and Tables

**Table 1:** Patients' baseline demographic and clinical characteristics

Variables	Patients (n = 96)
Age (years), median (range)	60.7 (33.3-83.1)
Sex/male, n (%)	81 (84.4)
BCLC stage, n (%)	
A	6 (6.3)
B	19 (19.8)
C	71 (74.0)
MVI, n (%)	47 (49.0)
VP stage, n (%)	
VP2	3 (6.4)
VP3	26 (55.3)
VP4	18 (38.3)
Out of up-to-7 criteria, n (%)	75 (78.1)
Out of up-to-11 criteria, n (%)	55 (57.3)
EHM, n (%)	45 (46.9)
Prior LRT, n (%)	
TACE	51 (53.1)
RFA	27 (28.1)
Prior TKI, n (%)	40 (41.7)
HBV history, n (%)	96 (100.0)
Baseline NLR, median (range)	3.9 (1.4-51.4)
Baseline platelets (10^3^/μL), median (range)	176.0 (39.0-750.0)
Baseline albumin (g/dL), median (range)	3.8 (2.5-4.7)
Baseline AFP (ng/mL), median (range)	371.0 (2.0-211754.9)
Baseline γ-GT (U/L), median (range)	127.0 (19.0-768.0)
Baseline ALP (U/L), median (range)	119.0 (44.0-1283.0)
Baseline ALBI II+III, n (%)	61 (63.5)
Previous gastrointestinal drug, n (%)	16 (16.7)
GALNT14-rs9679162, n (%)	
Non-GG	75 (78.1)
GG	21 (21.9)
GALNT14-rs6752303, n (%)	
Non-TT	73 (76.0)
TT	23 (24.0)
BMP7-rs6025211, n (%)	
Non-TT	82 (85.4)
TT	14 (14.6)
WWOX-rs13338697, n (%)	
Non-GG	87 (90.6)
GG	9 (9.4)
WWOX-rs13333314, n (%)	
Non-AA	87 (90.6)
AA	9 (9.4)

Abbreviations: ICI, immune checkpoint inhibitor; BCLC; Barcelona Clinic Liver Cancer classification; MVI, microvascular invasion; VP, portal vein invasion; EHM, extra-hepatic metastasis; TACE, transarterial chemoembolization; RFA, radiofrequency ablation; HBV, hepatitis B virus; NLR, neutrophil-to-lymphocyte ratio; AST, aspartate aminotransferase; ALT, alanine transaminase; AFP, alpha fetoprotein; ALBI grade, albumin-bilirubin grade; APRI, aspartate aminotransferase to platelet ratio index; FIB-4, fibrosis-4 index; LRT, locoregional therapy; IQR, interquartile range. ^a^Transarterial chemoembolization or radiofrequency ablation. *p < 0.05.

**Table 2:** Patients' response and outcome assessment for immune therapy between the two genotypes.

	GALNT14- rs9679162					GALNT14-rs6752303				
Review according to mRECIST	Non-GG (n = 75)		GG (n = 21)		p-value	Non-TT (n = 73)	TT (n = 23)			p-value
AFP decrease 20% from baseline^a^, n (%)	20 (26.7)		7 (33.3)		0.588	20 (27.4)	7 (30.4)			0.794
Treatment response, n (%)					0.210					0.166
Complete response	0 (0)		(0)			0 (0)	(0)			
Partial response	15 (20.0)		5 (23.8)			15 (20.5)	5 (21.7)			
Stable disease	23 (30.7)		10 (47.6)			22 (30.1)	11 (47.8)			
Progressive disease	27 (36.0)		6 (28.6)			26 (35.6)	7 (30.4)			
Not evaluable	10 (13.3)		0 (0)			10 (13.7)	0 (0)			
Objective response rate, n (%)	15 (20.0)		5 (23.8)		0.763	15 (20.5)	5 (21.7)			1.000
Disease control rate, n (%)	38 (50.7)		15 (71.4)		0.136	37 (50.7)	16 (69.6)			0.150
Follow up duration (months), median (range)	7.8 (0.1, 34.77)		10.4 (1.4, 33.4)		0.158	7.8 (0.1, 34.77)	10.4 (1.4, 33.4)			0.158
Overall survival (months), median (95% CI)	12.3 (7.2-17.3)		17.4 (4.4-32.8)		0.245	12.3 (10.0, 14.5)	17.4 (3.6, 31.2)			0.392
Progression-free survival (months), median (95% CI)	4.3 (0-9.9)		19.7 (12.3-35.7)		0.032*	4.3 (0, 9.8)	19.7 (5.2, 34.3)			0.056
										

Abbreviations: mRECIST, modified Response Evaluation Criteria in Solid Tumors; AFP, alpha fetoprotein. ^a^Patients with a baseline AFP level < 10 were excluded from AFP decrease by 20% analysis.

**Table 3:** Univariate and multivariate logistic regression analyses of post-treatment AST grade

Variables		Number	Univariate		Multivariate	
			OR (95% CI)	p-value	OR (95% CI)	p-value
Sex	Female	15	Ref.		Ref.	
	Male	81	0.673 (0.419, 3.852)	0.673	0.703 (0.224, 2.205)	0.546
Age		96	0.985 (0.949, 1.023)	0.436	0.990 (0.953, 1.028)	0.591
GALNT14_rs9679162	Non-GG	75	Ref.		Ref.	
	GG	21	0.284 (0.092, 0.880)	0.029*	0.298 (0.096, 0.926)	0.036*
BCLC stage	A	6	Ref.			
	B	19	7.857 (0.452, 82.128)	0.185		
	C	71	5.469 (0.606, 49.351)	0.130		
MVI	No	49	Ref.			
	Yes	47	0.737 (0.323, 1.683)	0.469		
Out of up-to-7 criteria	No	21	Ref.			
	Yes	75	1.583 (0.592, 4.235)	0.360		
EHM	No	51	Ref.			
	Yes	45	1.495 (0.654, 3.419)	0.340		
Prior LRT	No	40	Ref.			
	Yes	56	0.530 (0.226, 1.247)	0.146		
Prior TKI	No	56	Ref.			
	Yes	40	0.844 (0.367, 1.946)	0.691		
ALBI	I	35	Ref.			
	II+III	61	0.545 (0.228, 1.303)	0.172		
AFP drop 20% from baseline	No	69	Ref.			
	Yes	27	0.684 (0.236, 1.982)	0.484		

AST, aspartate amino transferase; BCLC; Barcelona Clinic Liver Cancer classification; MVI, microvascular invasion; EHM, extra-hepatic metastasis; LRT, loco-regional therapy; TKI, tyrosine kinase inhibitor; ALBI grade, albumin-bilirubin grade; AFP, alpha fetoprotein; OR, odds ratio; CI, confidence interval. *p < 0.05.

**Table 4:** Univariate and multivariate Cox regression analyses of post-treatment disease progression.

Variables		Number	Univariate		Multivariate	
			HR (95% CI)	p-value	HR (95% CI)	p-value
Sex	Female	15	Ref.		Ref.	
	Male	81	0.654 (0.336, 1.273)	0.211	0.821 (0.418, 1.612)	0.566
Age		96	0.976 (0.954, 0.997)	0.040*	0.979 (0.956, 0.999)	0.048*
GALNT14_ rs9679162	Non-GG	75	Ref.			
	GG	21	0.468 (0.230, 0.952)	0.036*	0.433 (0.212, 0.886)	0.022*
BCLC stage	A	6	Ref.			
	B	19	2.631 (0.578, 11.967)	0.211		
	C	71	2.845 (0.688, 11.758)	0.149		
MVI	No	49	Ref.			
	Yes	47	1.310 (0.787, 2.181)	0.298		
Out of up-to-7 criteria	No	21	Ref.			
	Yes	75	1.407 (0.745, 2.654)	0.292		
EHM	No	51	Ref.			
	Yes	45	1.010 (0.608, 1.678)	0.969		
Prior LRT	No	40	Ref.			
	Yes	56	0.970 (0.582, 1.617)	0.907		
Prior TKI	No	56	Ref.			
	Yes	40	1.375 (0.824, 2.293)	0.233		
ALBI	I	35	Ref.		Ref.	
	II+III	61	1.913 (1.087, 3.368)	0.025*	2.053 (1.153, 3.590)	0.014*

AST, aspartate amino transferase; BCLC; Barcelona Clinic Liver Cancer classification; MVI, microvascular invasion; EHM, extra-hepatic metastasis; ALBI grade, albumin-bilirubin grade; AFP, alpha fetoprotein; OR, odds ratio; CI, confidence interval. *p < 0.05.

**Table 5:** Multivariate Cox regression analyses of post-treatment disease progression after stratifying for AST grade.

Variables	Grade 0	p-value	Grade ≥ 1	p-value
	HR (95% CI)		HR (95% CI)	
Age	0.996 (0.961, 1.032)	0.827	0.968 (0.936, 1.002)	0.065
GALNT14_rs9679162				
Non-GG	Ref.			
GG	0.350 (0.132, 0.929)	0.035*	0.359 (0.046, 2.779)	0.327
ALBI				
I	Ref.		Ref.	
II+III	1.711 (0.732, 3.999)	0.215	2.523 (1.122, 5.672)	0.025*

AST, aspartate amino transferase; ALBI grade, albumin-bilirubin grade; AFP, alpha fetoprotein; HR, hazards ratio; CI, confidence interval. *p < 0.05.

## Abbreviations

AST: aspartate aminotransferase
DCR: disease control rate
HCC: hepatocellular carcinoma
HFSR: hand-foot skin reaction
ICIs: immune checkpoint inhibitors
ORR: objective response rate
OS: overall survival
PFS: progression-free survival
SNP: single nucleotide polymorphism
