# Supplementary material for: GALNT14-rs9679162 Genotypes Predict Post-immunotherapy Side Effect and Survival in Patients with Hepatitis B Virus-related Hepatocellular Carcinoma
Source: J Cancer. 2026 Jun 10;17(6):1220–8. doi: 10.7150/jca.133473 (PMC13280630; doi:10.7150/jca.133473)

Supplementary Table 1. Patients' response and outcome assessment for immune therapy between the two genotypes.

	BMP7-rs6025211			*WWOX*- rs13338697			*WWOX*- rs13333314		
**Review according to mRECIST**	**Non-TT (n = 82)**	**TT (n = 14)**	**p-value**	**Non-GG (n = 87)**	**GG (n = 9)**	**p-value**	**Non-AA (n = 87)**	**AA (n = 9)**	**p-value**
**AFP decrease 20% from baseline**[a], n (%)	23 (28.0)	4 (28.6)	1.000	24 (27.6)	3 (33.3)	1.000	24 (27.6)	3 (33.3)	1.000
**Treatment response**, n (%)			0.132			1.000			1.000
Complete response	0 (0)	(0)		0 (0)	(0)		0 (0)	(0)	
Partial response	18 (22.0)	2 (14.3)		18 (20.7)	2 (22.2)		18 (20.7)	2 (22.2)	
Stable disease	26 (31.7)	7 (50.0)		30 (34.5)	3 (33.3)		30 (34.5)	3 (33.3)	
Progressive disease	31 (37.8)	2 (14.3)		30 (34.5)	3 (33.3)		30 (34.5)	3 (33.3)	
Not evaluable	7 (8.5)	3 (21.4)		9 (10.3)	1 (11.1)		9 (10.3)	1 (11.1)	
**Objective response rate**, n (%)	18 (22.0)	2 (14.3)	1.000	18 (20.7)	2 (22.2)		18 (20.7)	2 (22.2)	
**Disease control rate**, n (%)	44 (53.7)	9 (64.3)	0.567	48 (55.2)	5 (55.6)	1.000	48 (55.2)	5 (55.6)	1.000
**Follow up duration (months)**, median (range)	8.8 (0.3, 34.8)	8.1 (0.2, 33.7)	0.934	8.0 (0.1, 34.8)	12.0 (1.1, 27.1)	0.447	8.0 (0.1, 34.8)	12.0 (1.1, 27.1)	0.447
**Overall survival (months)**, mean (95% CI)	17.2 (14.0, 20.4)	21.5 (13.3, 29.7)	0.381	18.5 (15.2-21.8)	12.7 (7.7-17.7)	0.408	18.5 (15.2-21.8)	12.7 (7.7-17.7)	0.408
**Progression-free survival (months)**, mean (95% CI)	13.0 (9.9, 16.0)	19.6 (11.4, 27.9)	0.159	14.3 (11.2, 17.4)	10.4 (4.4, 16.4)	0.571	14.3 (11.2, 17.4)	10.4 (4.4, 16.4)	0.571

Abbreviations: mRECIST, modified Response Evaluation Criteria in Solid Tumors; AFP, alpha fetoprotein. [a]Patients with a baseline AFP level < 10 were excluded from AFP decrease by 20% analysis.

Supplementary Table 2. Side effects in patients with the two *GALNT14* genotypes.

	rs6752303		p-value	rs9679162		p-value
	**Non-TT (n = 73)**	**TT (n = 23)**		**Non-GG (n = 75)**	**GG (n = 21)**	
**AST (grade)**, n (%)			0.010*			0.034*
0	29 (40.8)	15 (75.0)		31 (42.5)	13 (72.2)	
≥ 1	42 (59.2)	5 (25.0)		42 (57.5)	5 (27.8)	
**ALT (grade)**, n (%)			0.135			0.301
0	36 (51.4)	15 (71.4)		38 (52.8)	13 (68.4)	
≥ 1	34 (48.6)	6 (28.6)		34 (47.2)	6 (31.6)	
**Hypertension (grade)**, n (%)			0.228			0.193
0	62 (84.9)	17 (73.9)		64 (85.3)	15 (71.4)	
≥ 1	11 (15.1)	6 (26.1)		11 (14.7)	6 (28.6)	
**Proteinuria (grade)**, n (%)			1.000			0.776
0	55 (75.3)	17 (73.9)		57 (76.0)	15 (71.4)	
≥ 1	18 (24.7)	6 (26.1)		18 (24.0)	6 (28.6)	
**Hyperthyroidism (grade)**, n (%)			1.000			1.000
0	72 (98.6)	23 (100.0)		74 (98.7)	21 (100.0)	
≥ 1	1 (1.4)	0 (0)		1 (1.3)	0 (0)	
**Hypothyroidism (grade)**, n (%)			0.672			0.645
0	68 (93.2)	21 (91.3)		70 (93.3)	19 (90.5)	
≥ 1	5 (6.8)	2 (8.7)		5 (6.7)	2 (9.5)	
**Rash (grade)**, n (%)			1.000			1.000
0	66 (90.4)	21 (91.3)		68 (90.7)	19 (90.5)	
≥ 1	7 (9.6)	2 (8.7)		7 (9.3)	2 (9.5)	
**Fatigue (grade)**, n (%)			1.000			0.757
0	60 (82.2)	19 (82.6)		61 (81.3)	18 (85.7)	
≥ 1	13 (17.8)	4 (17.4)		14 (18.7)	3 (14.3)	
**Dizziness (grade)**, n (%)			0.590			0.300
0	70 (95.9)	21 (91.3)		72 (96.0)	19 (90.5)	
≥ 1	3 (4.1)	2 (8.7)		3 (4.0)	2 (9.5)	
**Myalgia (grade)**, n (%)			0.672			1.000
0	68 (93.2)	21 (91.3)		69 (92.0)	20 (95.2)	
≥ 1	5 (6.8)	2 (8.7)		6 (8.0)	1 (4.8)	
**Pruritus (grade)**, n (%)			1.000			1.000
0	66 (90.4)	21 (91.3)		68 (90.7)	19 (90.5)	
≥ 1	7 (9.6)	2 (8.7)		7 (9.3)	2 (9.5)	
**Diarrhea (grade)**, n (%)			1.000			1.000
0	64 (87.7)	21 (91.4)		66 (88.0)	19 (90.5)	
≥ 1	9 (12.3)	2 (8.7)		9 (12.0)	2 (9.5)	
**HFSR (grade)**, n (%)			1.000			1.000
0	70 (95.9)	22 (95.7)		72 (96.0)	20 (95.2)	
≥ 1	3 (4.1)	1 (4.3)		3 (4.0)	1 (4.8)	
**Edema (grade)**, n (%)			0.627			0.609
0	69 (94.5)	21 (91.3)		71 (94.7)	19 (90.5)	
≥ 1	4 (5.5)	2 (8.7)		4 (5.3)	2 (9.5)	
**Fever (grade)**, n (%)			0.147			0.229

0	56 (76.7)	21 (91.3)		58 (77.3)	19 (90.5)	
≥ 1	17 (23.3)	2 (8.7)		17 (22.7)	2 (9.5)	
**Pneumonitis (grade)**, n (%)			1.000			1.000
0	72 (98.6)	23 (100.0)		74 (98.7)	21 (100.0)	
≥ 1	1 (1.4)	0 (0)		1 (1.3)	0 (0)	
**Colitis (grade)**, n (%)			1.000			1.000
0	71 (97.3)	23 (100.0)		73 (97.3)	21 (100.0)	
≥ 1	2 (2.7)	0 (0)		2 (2.7)	0 (0)	
**Abdominal pain (grade)**, n (%)			0.145			0.070
0	60 (82.2)	15 (65.2)		62 (82.7)	13 (61.9)	
≥ 1	13 (17.8)	8 (34.8)		13 (17.3)	8 (38.1)	
**Vomiting + nausea (grade)**, n (%)			1.000			1.000
0	67 (91.8)	22 (95.7)		69 (92.0)	20 (95.2)	
≥ 1	6 (8.2)	1 (4.3)		6 (8.0)	1 (4.8)	
**GI bleeding (grade)**, n (%)			0.721			0.701
0	65 (89.0)	20 (87.0)		67 (89.3)	18 (85.7)	
≥ 1	8 (11.0)	3 (13.0)		8 (10.7)	3 (14.3)	
**GI bleeding prevention (grade)**, n (%)			0.672			0.645
0	68 (93.2)	21 (91.3)		70 (93.3)	19 (90.5)	
≥ 1	5 (6.8)	2 (8.7)		5 (6.7)	2 (9.5)	

AST, aspartate amino transferase; ALT, alanine amino transferase; HFSR, hand-foot skin reaction; GI, gastrointestinal.

Supplementary Table 3. Univariate and multivariate logistic regression analyses of post-treatment AST grade

Variables		Number	Univariate		Multivariate	
			**OR (95% CI)**	**p-value**	**OR (95% CI)**	**p-value**
**Sex**	Female	15	Ref.		Ref.	
	Male	81	0.788 (0.260, 2.389)	0.673	0.741 (0.233, 2.359)	0.612
**Age**		96	0.985 (0.949, 1.023)	0.436	0.989 (0.952, 1.028)	0.585
***GALNT14*-rs6752303**	Non-TT	73	Ref.		Ref.	
	TT	23	0.230 (0.075, 0.703)	0.010*	0.236 (0.077, 0.721)	0.011*
**BCLC stage**	A	6	Ref.			
	B	19	7.857 (0.352, 82.128)	0.185		
	C	71	5.469 (0.606, 49.351)	0.130		
**MVI**	No	49	Ref.			
	Yes	47	0.737 (0.323, 1.683)	0.467		
**Out of up-to-7 criteria**	No	21	Ref.			
	Yes	75	1.583 (0.592, 4.235)	0.360		
**EHM**	No	51	Ref.			
	Yes	45	1.495 (0.654, 3.419)	0.340		
**Prior LRT**	No	40	Ref.			
	Yes	56	0.530 (0.226, 1.247)	0.146		
**Prior TKI**	No	55	Ref.			
	Yes	40	0.691 (0.367, 1.946)	0.691		
**ALBI**	I	35	Ref.			
	II+III	61	0.545 (0.228, 1.303)	0.172		

AST, aspartate amino transferase; BCLC; Barcelona Clinic Liver Cancer classification; MVI, microvascular invasion; EHM, extra-hepatic metastasis; ALBI grade, albumin-bilirubin grade; AFP, alpha fetoprotein; OR, odds ratio; CI, confidence interval. *$p < 0.05$.

Supplementary Figure 1 Flowchart of patient inclusion. Abbreviations: HCC, hepatocellular carcinoma; ICI, immune checkpoint inhibitor.

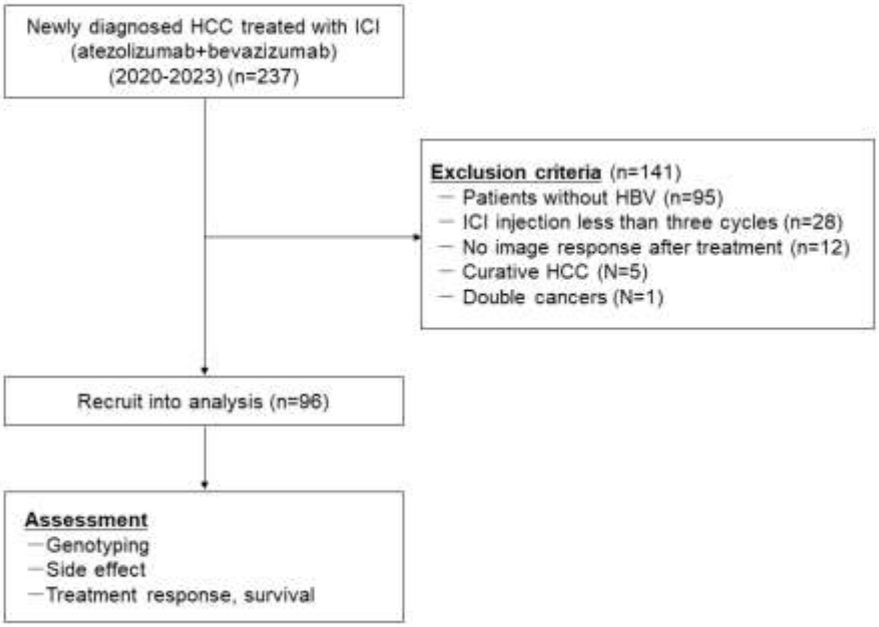

Supplement: Supplementary file 1 — Supplementary tables. [file jcav17p1220s1.pdf]
